# New perspective on first-trimester serum uric acid level in predicting the risk of gestational diabetes mellitus

**DOI:** 10.1038/s41598-024-51507-8

**Published:** 2024-01-08

**Authors:** Xiaojing Li, Ziru Niu, Liwei Bai, Qiang Lu

**Affiliations:** 1 Department of Endocrinology, First Hospital of Qinhuangdao, Hebei, 066000 Qinhuangdao China; 2 Department of Obstetrics, Qinhuangdao Hospital for Maternal and Child Health, Hebei, 066000 Qinhuangdao China; 3 Department of Obstetrics, First Hospital of Qinhuangdao, Hebei, 066000 Qinhuangdao China

**Keywords:** Risk factors, Diabetes

## Abstract

This study aimed to investigate the correlation between serum uric acid (UA) and gestational diabetes mellitus (GDM) during the first trimester and provide a new perspective for the prevention and treatment of GDM. Based on the diagnostic criteria of gestational diabetes of the International Association of Diabetes and Pregnancy Study Groups, 1744 and 4256 patients were enrolled in the GDM and normal glucose tolerance (NGT) groups. Four groups were constituted based on the quartile of first-trimester serum UA (UA) level, and the differences in each indicator between groups were compared. Logistic regression was used to analyze the effects of UA level on GDM risk. The rate of GDM in the UA quartile changed from low to high. Significant differences were also observed in fasting plasma glucose level, 1 h post glucose and 2 h post glucose levels, in all the groups (P < 0.05), which increased with the UA level. UA level were independent risk factors for GDM. The best threshold of GDM predicted by the first-trimester UA level was 226.55 μmol/L. The first-trimester UA level in patients with GDM was relatively higher and was an independent risk factor for GDM.

## Introduction

Gestational diabetes mellitus (GDM) is one of the most common pregnancy complications, with adverse effects on both the mother and the fetus^1^. Besides the traditional risk factors of GDM, for example, maternal obesity, advanced maternal age, and family history of diabetes^2^, some new risk factors have also been gradually discovered and paid attention to. Of these, uric acid (UA) is considered a potential indicator that predicts the risk of pregnancy-related adverse outcomes, such as GDM. The UA level has been reported to be higher in women with GDM than in pregnant women with normal glucose tolerance (NGT) and may participate in the pathogenesis of GDM with adverse effects on maternal and neonatal outcomes^3^. Earlier (First-Trimester) screening may improve GDM-associated outcomes^4^. Therefore, currently new methods of early screening of GDM are in the focus of scientific and clinical research. Currently there are several early GDM screening approaches such as prediction model approach, ultrasound approach and biochemical approach^5–7^. A few large-scale studies exist to date on the correlation between UA and GDM. This study retrospectively analyzed the association between UA and GDM in 6000 pregnant women in the first trimester, and evaluated the ability of indicators to predict GDM, providing a new perspective on the early warning for preventing GDM.

## Methods

### Study participants

This study is a cross-sectional observation study. A total of 6000 pregnant women undergoing routine prenatal examination in the Qinhuangdao Maternal and Child Health Hospital from January 2016 to January 2022 were analyzed. The results of oral glucose tolerance test (OGTT) after 24–28 weeks of gestation were collected. The study participants were categorized into the NGT (*n* = 4256) and GDM groups (*n* = 1744) based on OGTT. This study was approved by the ethics committees of the First Hospital of Qinhuangdao and the Qinhuangdao Maternal and Child Health Hospital.

### Measurement of laboratory and other test parameters

Age, height, prepregnancy weight, pregnancy weight, and so on were recorded, and body mass index (BMI) was calculated. The laboratory test results from 8 to 12 weeks of gestation were obtained, including the levels of glycosylated hemoglobin (HbA1c), TG, total cholesterol (TC), high-density lipoprotein cholesterol (HDL-C), low-density lipoprotein cholesterol (LDL-C), UA, blood urea nitrogen (BUN), creatinine (CREA), alanine aminotransferase (ALT), aspartate aminotransferase (AST), γ-glutamyltransferase (GGT), and so forth.

### Diagnostic criteria for GDM

Based on the diagnostic criteria of the International Association of Diabetes and Pregnancy Study Groups (IADPSG)^22^, GDM is diagnosed if any one of the following three criteria is met: levels of fasting plasma glucose (FPG) ≥ 5.1 mmol/L, 1 h post glucose (1hPG) ≥ 10.0 mmol/L, and 2 h post glucose (2hPG) ≥ 8.5 mmol/L.

### Exclusion criteria

The exclusion criteria were as follows: (1) diabetes mellitus combined with pregnancy and overt gestational diabetes; (2) hypertension; (3) thyroid diseases, abnormal liver, and kidney function; (4) other chronic diseases and complications of pregnancy; (5) recent history of acute infection; and (6) pregnant women with incomplete medical records.

### Statistical analysis

SPSS Statistics 25.0 software was used for statistical analysis. The pregnant women in the GDM and NGT groups were selected for analysis. The measurement data were expressed as (*x* ± *s*), and a two-sample *t* test was used for comparison between groups. The enumeration data were expressed as [example (%)], and the *χ*^2^ test was conducted to compare between groups. A *P* value < 0.05 indicated a statistically significant difference. Logistic regression was performed to analyze the association between meaningful indicators and GDM, and the receiver operating characteristic (ROC) curve was used to evaluate the ability of indicators to predict GDM.

### Ethics approval and consent to participate

I confirm that all methods were performed in accordance with the relevant guidelines. This work has been carried out in accordance with the Declaration of Helsinki (2000) of the World Medical Association. This study was approved by the Ethics Committee of Qinhuangdao First Hospital, the Ethics Committee of Qinhuangdao Maternal and Child Health Care Hospital[2021Q088]. The requirement for informed consent was waived by the Institutional Review Board of Ethics Committee of Qinhuangdao First Hospital, the Ethics Committee of Qinhuangdao Maternal and Child Health Care Hospital because of the retrospective nature of the study.

## Results

### Comparison of general data and laboratory results between two groups

#### Comparison of general data

The age of the 6000 patients was recorded as 29.51 ± 4.13 years, ranging between 17 and 50 years. Differences were observed in age, height, prepregnancy weight, pregnancy weight, and prepregnancy BMI between the GDM and the NGT groups (*P* < 0.05), but no significant differences were observed in weight gain during the second trimester (Table 1).

**Table 1:** Comparison of general data between two groups.

Group	Number of patients	Age	Height	Prepregnancy weight	Pregnancy weight	BMI	Weight gain
NGT	4256	29.15 ± 4.03	162.84 ± 4.21	58.18 ± 8.66	64.19 ± 9.16	21.94 ± 3.14	6.01 ± 4.09
GDM	1744	30.39 ± 4.25	162.43 ± 4.56	61.97 ± 10.54	68.08 ± 10.54	23.48 ± 3.79	6.11 ± 3.78
*t*	–	− 10.373	3.252	− 13.320	− 13.480	− 15.023	− 0.883
*P*	–	< 0.001	0.001	< 0.001	< 0.001	< 0.001	0.393

#### Comparison of glycolipid metabolism and biochemical indicators between groups

The levels of HbA1c, FPG, OGTT 1hPG, 2hPG, TG, HDL-C, and LDL-C were higher in the GDM group than in the NGT group (*P* < 0.05), but no significant difference was observed in the TC level between the two groups (*P* ≥ 0.05) (Table 2). The levels of ALT, GGT, UA, BUN, CREA, and TG/HDL-C were higher in the GDM group than in the control group (*P* < 0.05), but no significant difference was observed in AST level between the two groups (*P* ≥ 0.05) (Table 3).

**Table 2:** Comparison of glycolipid levels between two groups.

Group	Number of patients	HbA1c	FPG	1hPG	2hPG	TG	TC	HDL-C	LDL-C
NGT	4256	4.90 ± 0.29	4.53 ± 0.33	7.20 ± 1.23	6.24 ± 0.98	1.51 ± 0.64	4.63 ± 0.89	1.99 ± 0.49	1.88 ± 0.64
GDM	1744	5.15 ± 0.32	5.20 ± 0.50	9.33 ± 1.64	7.88 ± 1.44	1.82 ± 0.79	4.63 ± 0.86	1.83 ± 0.46	1.96 ± 0.69
*t*	–	− 28.296	− 51.907	− 48.745	− 43.446	− 14.278	− 0.055	12.496	− 4.087
*P*	–	< 0.001	< 0.001	< 0.001	< 0.001	< 0.001	0.957	< 0.001	< 0.001

**Table 3:** Comparison of UA and other biochemical indicators between two groups.

Group	Number of patients	UA	ALT	AST	GGT	BUN	CREA	TG/HDL-C
NGT	4256	201.92 ± 45.29	14.78 ± 14.40	17.58 ± 8.79	12.86 ± 10.58	3.11 ± 2.57	44.34 ± 7.14	0.81 ± 0.48
GDM	1744	240.84 ± 63.19	15.73 ± 16.09	17.64 ± 8.79	14.79 ± 10.47	3.58 ± 3.98	44.83 ± 7.88	1.07 ± 0.63
*t*	–	− 5.797	− 2.142	− 0.261	− 6.448	− 4.564	− 2.228	− 15.816
*P*	–	< 0.001	0.032	0.794	< 0.001	< 0.001	0.026	< 0.001

#### Basic information on UA quartile

A total of 6000 patients, including 1744 patients with GDM and 4256 patients with NGT, were included in this study. The pregnant women were classified into four groups, groups Q1, Q2, Q3, and Q4, based on the first-trimester UA quartile. The mean UA values were 153.81 ± 18.22, 191.60 ± 8.62, 222.44 ± 10.03, and 285.41 ± 43.72 μmol/L in groups Q1–Q4, respectively. The GDM ratios were 15.2% (228/1275) in group Q1, 23.0% (346/1158) in group Q2, 28.1% (420/1076) in group Q3, and 50.1% (750/747) in group Q4, suggesting that the GDM rate in the UA quartile changed from low to high (*P* < 0.001). Differences were observed in age, prepregnancy weight, pregnancy weight, and prepregnancy BMI between groups (*P* < 0.001), as well as in HbA1c, TG, TC, AST, ALT, GGT, and CREA levels and TG/HDL-C ratio in the first trimester (*P* < 0.001). Differences were also observed in FPG and OGTT 1hPG and 2hPG levels in the second trimester (*P* < 0.001), which increased with the UA level. No significant differences were observed in the weight gain and levels of urea, HDL-C, and LDL-C during pregnancy between groups (*P* ≥ 0.05) (Table 4).

**Table 4:** Comparison of clinical data based on first-trimester UA level (*x* ± *s*).

Group	Number of patients	GDM ratio	GDM/NGT	Age	Height	Prepregnancy weight	BMI	Pregnancy weight	Weight gain
Q1	1503	15.2%	228/1275	29.9 ± 4.18	162.68 ± 4.26	57.21 ± 8.24	21.62 ± 3.00	63.36 ± 8.75	6.15 ± 3.99
Q2	1504	23.0%	346/1158	29.3 ± 3.98	162.89 ± 4.40	58.76 ± 9.18	22.14 ± 3.29	64.85 ± 9.60	6.09 ± 4.07
Q3	1496	28.1%	420/1076	29.2 ± 4.01	162.80 ± 4.33	59.67 ± 9.34	22.51 ± 3.38	65.71 ± 9.67	6.04 ± 4.11
Q4	1497	50.1%	750/747	29.5 ± 4.31	162.51 ± 4.28	61.49 ± 10.24	23.28 ± 3.74	67.37 ± 10.45	5.89 ± 3.84
		< 0.001	< 0.001	< 0.001	0.088	< 0.001	< 0.001	< 0.001	0.311

Group	Number of patients	UA	Urea	CREA	HbA1c	FPG	1hPG	2hPG	
Q1	1503	153.81 ± 18.22	2.76 ± 1.10	43.56 ± 6.69	4.94 ± 0.31	4.63 ± 0.43	7.48 ± 1.45	6.45 ± 0.98	
Q2	1504	191.60 ± 8.62	2.80 ± 1.05	44.13 ± 6.94	4.96 ± 0.31	4.68 ± 0.44	7.65 ± 1.61	6.57 ± 1.44	
Q3	1496	222.44 ± 10.03	2.84 ± 1.04	44.80 ± 7.22	4.97 ± 0.32	4.73 ± 0.51	7.85 ± 1.68	6.75 ± 0.98	
Q 4	1497	285.41 ± 43.72	2.84 ± 0.93	44.59 ± 8.02	5.03 ± 0.34	4.86 ± 0.54	8.30 ± 1.82	7.11 ± 0.98	
		< 0.001	0.074	< 0.001	< 0.001	< 0.001	< 0.001	< 0.001	

Group	Number of patients	TG	TC	HDL-C	LDL-C	TG/HDL-C	ALT	AST	GGT
Q1	1503	1.45 ± 0.62	4.53 ± 0.85	1.94 ± 0.43	1.89 ± 0.64	0.79 ± 0.44	14.10 ± 12.76	16.96 ± 7.05	12.16 ± 7.49
Q2	1504	1.52 ± 0.62	4.62 ± 0.88	1.96 ± 0.47	1.92 ± 0.67	0.82 ± 0.41	14.47 ± 12.94	17.33 ± 7.77	12.89 ± 8.11
Q3	1496	1.60 ± 0.66	4.63 ± 0.86	1.95 ± 0.48	1.90 ± 0.64	0.89 ± 0.61	15.72 ± 14.09	17.75 ± 7.77	13.26 ± 7.51
Q4	1497	1.83 ± 0.82	4.74 ± 0.93	1.92 ± 0.55	1.91 ± 0.68	1.04 ± 0.62	15.90 ± 15.11	18.08 ± 9.10	15.13 ± 10.75
		< 0.001	< 0.001	0.168	0.592	< 0.001	< 0.001	0.001	< 0.001

#### Logistic regression analysis of the correlation between first-trimester UA and GDM

After correcting for age, prepregnancy BMI, first-trimester HbA1c, TG, and HDL-C data, the correlation between first-trimester UA and GDM risk was observed. Pregnant women with UA in the highest quartile had a 4.01 times higher risk of developing GDM than those in the lowest quartile (Table 5).

**Table 5:** Logistic regression analysis of correlation between first-trimester UA and GDM.

Group	Model 1		Model 2		Model 3	
	Odds ratio (95% CI)	*P*	Odds ratio (95% CI)	*P*	Odds ratio (95% CI)	*P*
Q1	1		1		1	
Q2	1.671 (1.388–2.011)	< 0.001	1.683 (1.393–2.034)	< 0.001	1.681 (1.377–2.052)	< 0.001
Q3	2.183 (1.822–2.615)	< 0.001	2.166 (1.800–2.606)	< 0.001	2.124 (1.747–2.584)	< 0.001
Q4	5.615 (4.720–6.679)	< 0.001	5.270 (4.406–6.303)	< 0.001	5.010 (4.136–6.068)	< 0.001

Model 1 does not correct for confounding factors; Model 2 corrects for age and prepregnancy BMI; Model 3 corrects for first-trimester HbA1c, TG, and HDL-C based on the age and prepregnancy BMI. *CI* confidence interval.

#### Binary logistic regression analysis results

GDM was used as a dependent variable, and age, prepregnancy BMI, HbA1c, TG, HDL-C, LDL-C, TG/HDL-C, ALT, GGT, UA, CREA, and BUN were used as independent variables for binary logistic regression analysis. The results suggested that age, prepregnancy BMI, UA level, TG level, HDL-C level, and HbA1c level were significantly correlated with GDM (*P* < 0.001) (Table 6).

**Table 6:** Logistic regression analysis results.

Index	*β*	*P*	Odds ratio	95% CI
Age	0.059	< 0.001	1.061	1.045–1.078
BMI	0.036	< 0.001	1.036	1.016–1.057
HbA1c	2.437	< 0.001	11.434	9.044–14.457
UA	0.013	< 0.001	1.013	1.012–1.014
TG	0.289	< 0.001	1.335	1.215–1.466
HDL-C	− 0.566	< 0.001	0.568	0.493–0.653
Constant	− 17.952	< 0.001	0.000	–

#### Predictive levels of UA, TG, TG/HDL-C, and HbA1c for GDM

**Figure 1:**
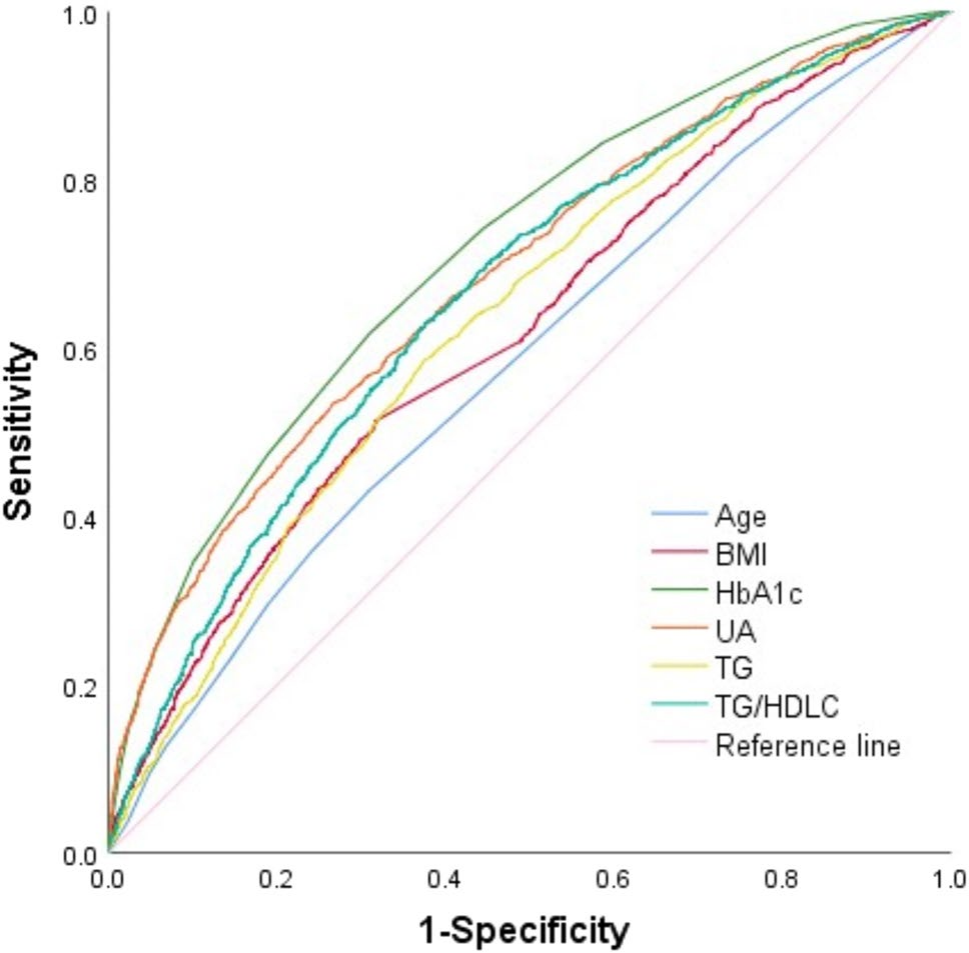
ROC curve of first-trimester UA, TG, TG/HDL-C, and HbA1c predicting the risk of GDM.

The predictive values of first-trimester UA, TG, TG/HDL-C, and HbA1c for GDM were analyzed using the ROC curve (Table 7 and Fig. 1).

**Table 7:** Predictive values of UA, TG, TG/HDL-C, and HbA1c for GDM.

Index	Best cutoff point	AUC	95% CI	Sensitivity (%)	Specificity (%)
Age	30.5	0.582	0.566–0.598	42.9	69.2
BMI	22.39	0.619	0.604–0.635	51.3	68.4
HbA1c	5.05	0.716	0.702–0.730	61.6	69.1
UA	226.55	0.685	0.670–0.700	53.3	73.5
TG	1.54	0.635	0.620–0.650	58.5	62.5
TG/HDL-C	0.81	0.665	0.650–0.680	62.9	62.5

## Discussion

UA is the end-product of purine nucleotide metabolism. UA at a physiological concentration induces a positive effect on antioxidation, free radical scavenging, and blood–brain barrier stability^8^. Nevertheless, too high a concentration of UA that induces hyperuricemia (HUA) is an independent risk factor for cardiovascular and cerebrovascular diseases, type 2 diabetes, and metabolic syndromes^9^. Similarly, it is an important predisposing factor for gestational insulin resistance (IR) and increases the risk of GDM^10^. Also a recent meta-analysis conducted on confirmed the role of serum acid uric as a potential independent risk factor for gestational diabetes mellitus (GDM)^11^. In a study including 24,023 pregnant women^12^, 3204 (13.44%) had GDM diagnosed between weeks 24 and 28 of pregnancy. UA levels earlier than 24 weeks of gestation were associated with the risk of GDM, and the association was closer at 18 weeks of gestation. Thus, UA detection before 18 weeks of gestation is recommended as the best time. UA was also related to preterm birth and GDM with preeclampsia in secondary outcomes. Li et al.^13^ reported that the UA level at 16–18 weeks of gestation was positively and independently correlated with the increased risk of GDM, and the highest UA quartile increased the risk by 55.7%. A cohort study^14^ including 85,609 pregnant women found that the elevated first-trimester UA level increased the risk of GDM, and emphasized the necessity to monitor UA after 13–18 weeks of gestation. Elevated first-trimester UA level has been proved to be associated with the development of GDM^15^. About one half of women with GDM (46.6%) had first-trimester UA levels in the highest quartile and had a 3.25-fold increased risk of developing GDM compared with those with the lowest quartile, which was consistent with our findings. Our study indicated that the UA level was significantly higher in the GDM group than in the NGT group. Based on the UA quartile grouping, the rate of GDM in each group also varied from high to low with the UA level. The risk of developing GDM was 4.01 times higher in women with the highest quartile than in those with the lowest quartile. In 50.1% of women with GDM, the first-trimester UA level was in the highest quartile, indicating that it was related to GDM. The aforementioned studies suggested that the first-trimester UA level was closely correlated with the occurrence of GDM, requiring more attention.

A number of studies have focused on the correlation between pregnancy UA and IR and impaired glucose metabolism. HUA enhances IR induced by oxidative stress and inflammatory cytokines, inevitably resulting in insulin dysfunction and abnormal glucose metabolism^16^. HUA-induced IR shares the same developmental and functional process in pregnant and nonpregnant women. Elevated UA levels may be correlated with IR and the increased risk of GDM. A prospective study on nonpregnancy^17^ suggested that HUA was a predisposing factor for IR and diabetes within 10 years, mostly in women. The UA level increased in pregnant women with significantly impaired glucose tolerance, indicating that UA level might be related to IR^18^. A previous study^19^ reported that the elevated UA level during the second trimester was associated with IR. Homeostasis model assessment for IR (HOMA-IR) index was positively correlated with the UA level and was more significant in pregnant women with BMI ≥ 25 kg/m^2^. This finding was also consistent with our study. We proved that the UA level increased from low to high in the quartile grouping, as did prepregnancy weight, prepregnancy, BMI, and pregnancy weight. GDM is usually the result of β-cell dysfunction induced by progressive IR during pregnancy. Hyperinsulinemic euglycemic clamp is a gold standard to evaluate IR, but it is mostly used in basic research because it is expensive and time-consuming. The use of HOMA-IR is not applicable in large populations due to its dependence on fasting insulin measurement. Recently, some simple IR assessment tools have emerged for assessing TG/HDL-C and triglyceride glucose (TyG) levels, which offer more options. A study from the United States explored the predictive value of four assessment indexes, such as TyG, TyG-BMI, TG/HDL-C, and metabolic score for IR, in patients with nondiabetic HUA. The result proved a good correlation with UA level^20^ and better clarified the correlation between IR and UA levels. The fasting insulin level was not measured in this study. IR could not be assessed with traditional HOMA-IR, but could be indirectly assessed with TG/HDL-C. It indicated that the TG/HDL-C ratio was significantly higher in the GDM group than in the normal group. In the UA quartile grouping, TG/HDL-C ratio also increased with the UA level, suggesting that IR gradually aggravated with the UA level. Although a large number of studies have reported that HUA may be an independent risk factor for IR, their causal relationship is controversial, possibly in terms of reciprocal causation.

Recently, the predictive abilities of 36 metabolites and 22 clinical indicators on the risk of GDM in the first trimester of pregnancy were analyzed^21^. The predictive potential of these serum phenotypes for GDM was as follows: eight phenotypes with an AUC > 0.68, including UA AUC of 0.71 and prepregnancy BMI AUC of up to 0.72. It further proved that UA was an independent risk factor for GDM. Some recent studies used UA and other indicators to assess GDM, which improved the assessment efficacy. In a study combining UA and blood lipids to predict adverse pregnancy outcomes during the second trimester^22^, ROC analysis revealed that the optimal cutoff of UA for predicting GDM was 225.5 μmol/L with the AUC of 0.58, sensitivity of 54%, and specificity of 65%. The sensitivity and specificity levels of a single marker were relatively low. However, when UA level, TG level, HDL-C level, age, and BMI were combined, the AUC increased to 0.71–0.77, and the sensitivity and specificity increased to 80–92% and 50–53%, respectively. In this study, maternal age, prepregnancy BMI, and first-trimester UA, HbA1c, TG, and HDL-C levels were independent risk factors with independent predictive values for GDM. The predictive ability of first-trimester HbA1c was relatively high, with an AUC of 0.716. The ability of UA was higher than those of age, prepregnancy BMI, and TG, with an AUC of 0.685. The optimal cutoff for predicting GDM was 226.55 μmol/L, with a sensitivity of 53.3% and a specificity of 73.5%. The predictive ability of the UA level and the optimal cutoff value were similar to the aforementioned test results. The use of UA for the early warning of GDM has received increasing attention. A study on the detection of saliva UA from pregnant women^23^ proved that saliva UA and UA levels could be used to detect UA levels in a large population, which is a noninvasive, convenient, low-cost method, and a new type of marker for predicting the risk during pregnancy in the future. As UA level fluctuates significantly during pregnancy, dynamic observation of the level before and during pregnancy is recommended. No uniform standard exists to date for the normal UA level during pregnancy and the starting point and target of treatment due to the lack of large-sample epidemiological data. The physiological concentration of UA in pregnant women after different weeks of gestation needs analysis^24^. Thus, UA levels should be closely monitored during pregnancy. A healthy lifestyle should be adopted, high purine and fructose intake should be avoided, excessive weight gain should be controlled, and regular exercise should be performed to prevent serious adverse maternal or neonatal outcomes^25^. In the clinic, the dynamic monitoring of UA-related indicators during pregnancy can be started in the first trimester of pregnancy. The emphasis on the basic value and increased rate of UA is crucial for early identification and intervention of pregnancy-related complications induced by elevated UA during pregnancy.

This study had some limitations. (1) This was a single-hospital and single-center clinical study including only pregnant women from one city in China and was a cross-sectional study. It could not represent the situation in multiple cities. (2) As UA is a dynamic indicator, this study might have information bias when one-measurement data were used. (3) Confounding factors such as diet and genes were not considered, which possibly impacted the overall assessment results.

Moreover, an elevated UA level induces a higher risk of GDM in pregnant women, and may even impact maternal and neonatal outcomes, as well as long-term prognosis. This study provided a new perspective for GDM prevention and early intervention, but the mechanism of UA during pregnancy is still unclear. Thus, we should pay attention to the UA variations in pregnant women, timely detection of UA abnormalities, and timely intervention. These may help reduce the occurrence of GDM and improve maternal or neonatal outcomes.

## Data Availability

The datasets used and/or analysed during the current study are available from the corresponding author on reasonable request.
